# Big data analysis for evaluating bioinvasion risk

**DOI:** 10.1186/s12859-018-2272-5

**Published:** 2018-08-13

**Authors:** Shengling Wang, Chenyu Wang, Shenling Wang, Liran Ma

**Affiliations:** 1 College of Information Science and Technology, Beijing Normal University, Beijing, 100875 China; 2 Department of Computer Science, Texas Christian University, Fort Worth, 298850 TX USA

**Keywords:** Bioinvasion, Species invasion network, *S*-core decomposition

## Abstract

**Background:**

Global maritime trade plays an important role in the modern transportation industry. It brings significant economic profit along with bioinvasion risk. Species translocate and establish in a non-native area through ballast water and biofouling. Aiming at aquatic bioinvasion issue, people proposed various suggestions for bioinvasion management. Nonetheless, these suggestions only focus on the chance of a port been affected but ignore the port’s ability to further spread the invaded species.

**Results:**

To tackle the issues of the existing work, we propose a biosecurity triggering mechanism, where the bioinvasion risk of a port is estimated according to both the invaded risk of a port and its power of being a stepping-stone. To compute the invaded risk, we utilize the automatic identification system data, the ballast water data and marine environmental data. According to the invaded risk of ports, we construct a species invasion network (SIN). The incoming bioinvasion risk is derived from invaded risk data while the invasion risk spreading capability of each port is evaluated by *s*-core decomposition of SIN.

**Conclusions:**

We illustrate 100 ports in the world that have the highest bioinvasion risk when the invaded risk and stepping-stone bioinvasion risk are equally treated. There are two bioinvasion risk intensive regions, namely the Western Europe (including the Western European margin and the Mediterranean) and the Asia-Pacific, which are just the region with a high growth rate of non-indigenous species and the area that has been identified as a source for many of non-indigenous species discovered elsewhere (especially the Asian clam, which is assumed to be the most invasive species worldwide).

## Background

### Introduction

Global maritime trade plays an essential part in people’s daily lives because many cargoes such as food, fuel, commodities are carried by vessels. According to the statistic from the United Nations Conference on Trade and Development (UNCTAD) [1], shipping contributes about 80% of global trade by volume and over 70% of global trade by value. However, the global maritime trade also accounts for aquatic bioinvasion. In detail, by way of discharging ballast water which may contain aquatic species from other ports passed by ships, the alien invasive species can be introduced. In addition, the hull fouling containing microorganisms, plants, algae, or animals is another major pathway to broadcast exotic aquatic species [2].

Once the marine species and viruses establish in a non-native region, they would cause massive damage in terms of lives and economy [3]. For example, in Europe, the economic loss of non-native terrestrial and aquatic species has been assessed to be at least € 12.5 billion per year and probably amounts to over € 20 billion [4]. At the same time, the control efforts including removal, prevention and management of marine invasive species also bring extra economic cost. Taking a macroalgae species that invaded Monterey Harbor as an example [5], the direct cost to remove this invasive species ran up to $160,300 for a 10-year period.

To address the issue of aquatic bioinvasion, one mainstream countermeasure is to propose suggestions for biomarker identification [6, 7] and bioinvasion management. However, the existing biosecurity suggestions [2, 8, 9] only considered the invaded risk of a port and neglected its role of being a *stepping-stone*, which means it can further spread the invaded species. The *stepping-stone* invasion should be paid more attention due to the relatively high proportion [10]. However, it is challenging to analyze a port’s power of further spreading the invaded aquatic species because the fluctuation of invaded risk in some ports may lead to *butterfly-effect* due to their special locations. Hence, the effect of *stepping-stone* should be analyzed from a global perspective.

To tackle the issues of existing work, a biosecurity triggering mechanism is proposed to instruct the biosecurity management. By our mechanism, some controls should be carried out when the bioinvasion risk exceeds a given threshold. We estimate the bioinvasion risk according to both the invaded risk of a port and its ability of further spreading invaded species. To compute the invaded risk of each route, we utilize the automatic identification system (AIS) data, the ballast water data and marine environmental data. According to the invaded risk of routes between any two ports, we construct a species invasion network (SIN). By manipulating *s*-core decomposition, we derived the *s*-shell value of each port, which is a significant metric to identify the port’s ability to further spread the bioinvasion risk since higher *s*-shell value indicates larger degree and more central position in SIN. Finally, we list 100 ports in the world that have the highest bioinvasion risk when the invaded risk and *stepping-stone* bioinvasion risk are equally treated. There are two bioinvasion risk intensive regions, namely the Western Europe (including the Western European margin and the Mediterranean) and the Asia-Pacific, which are just the region with a high growth rate of non-indigenous species and the area that has been identified as a source for many of non-indigenous species discovered elsewhere (especially the Asian clam, which is assumed perhaps the most invasive species worldwide).

### Related work

It is high time that bioinvasion should be addressed due to its negative impact on the ecosystem, society and economy. Currently, there exist two categories of mainstream countermeasures: the first is constructing different invasion threat assessment models [11–14] while the second is providing the suggestion for bioinvasion management [2, 8, 9]. Actually, some bioinvasion management suggestions were given according to some invasion threat assessment models. That is to say, two categories of countermeasures are not totally independent.

To estimate the invasion risk of alien species, various invasion threat assessment models [11–14] were built. To give advices on introducing new species to a native ecosystem, [11] developed a threat scoring framework to evaluate the invasion threat of each alien species to native biodiversity, and assessed the threat level of different invasive pathways. [12] established a risk model according to the number of ship visits and the environmental factor, so that it can figure out shipping routes that have a high probability to pour invasive species into a given port and the possible source regions. The probability of invasive species establishment in a marine region was computed in [13]. The aim was to provide a judgement basis for bioinvasion, where a biosecurity strategy could be triggered once such probability is greater than a given threshold. [14] developed the corresponding models to describe the probability of a species to be alien, the probabilities that a species can be introduced to and established in a given marine region. Such models were used to assess the invasion risk of ports and shipping routes.

Based on the idea of [14], [2] established a species flow network (SFN), from which the authors discovered invasion patterns through clustering analysis and then devised invasive species management strategies. [8] identified hot spots fragile to alien aquatic invasion according to worldwide patterns of ship traffic. The rate of port-to-port invasion was estimated using gravity models for spatial interactions, which helped to figure out bottlenecks to the regional exchange of species using the Ford-Fulkerson algorithm for network flows. In [9], two risk models, namely bioregion pathway and species-based exposure, were examined with the aim to determine an effective strategy to implement marine biosecurity risk management in regions/countries where biological data are limited.

Conclusively, the existing work [2, 8, 9] did not consider the invaded risk of a port and its power of spreading species at the same time, which is not enough to control the bioinvasion. We utilize the big data technology [15, 16] to tackle the current bioinvasion issue, based on which a species invasion network (SIN) is constructed. By *s*-core(*s*-shell) decomposition, developed from *k*-core(*k*-shell) decomposition, we calculate the level of popularity of each node in SIN. *k*-core decomposition is widely used in network analysis. [17] concentrates on the topology of the internet and separate the internet structure into three part by *k*-shell (*k*-core) decomposition method. [18] targets on large-scale software system and analyzes the software structure by utilizing *k*-shell (*k*-core) decomposition method. Taking the weight of edges into consideration, the *s*-core(*s*-shell) decomposition can be derived [19].

## Methods

### Framework and data

**Fig. 1:**
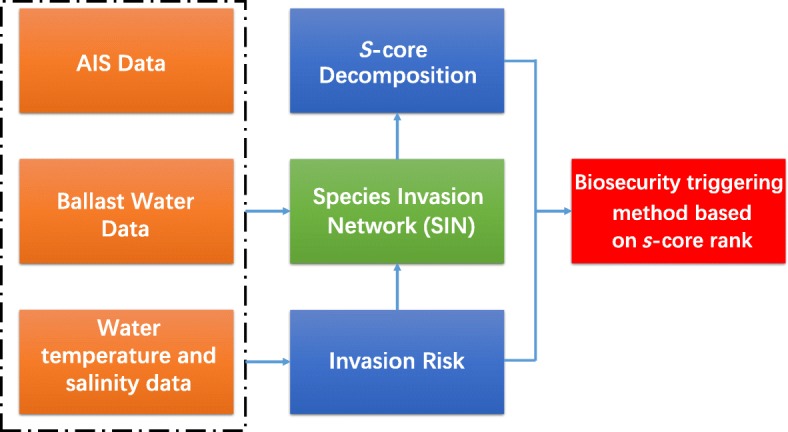
The framework of our analysis

The framework of our analysis is depicted in Fig. 1. We aim to evaluate the bioinvasion risk of major ports throughout the world. Intuitively, the bioinvasion risks of ports consist of the incoming one and the outgoing one.

A port’s incoming risk is estimated by aggregating invasion risk of all shipping routes passing through it. Conclusively, to calculate the invasion risk from one port to another, we need three kinds of information, that is, shipping information (including shipping routes passing through each port in the world, the corresponding travel time and status), the ballast water information and the marine environmental information (including water temperature and salinity). Hence, we take advantage of 12-month AIS data in 2014 to obtain the shipping information. The data includes 234,661,079 records and each piece of record provides the following information: the feedback time of GPS, the shipping status (its value ranges from 0-15. Specifically, 1 = the ship is anchored, 2 = the ship is not in operation, 7= the ship is doing fishery, and 8 = the ship is sailing), the longitude and the latitude of the anchorage, and the official number of each ship which is used to identify one ship uniquely. Besides, we obtain the ballast water information from National Ballast Information Clearinghouse (NBIC), using its ballast discharge data ranging from 2004-01-01 to 2016-08-25 for our analysis, which contains 1,080,456 records of all ships visiting the ports of USA. Finally, we employ the marine environmental information from the World Ocean Atlas 2013 version 2 [20] to obtain the water temperature and salinity for any given ports.

To derive the outgoing risk, we set up a species invasion network (SIN), which is constructed by combining the global maritime trade trajectory and the invasion risks of routes. By employing the *s*-core decomposition of SIN, we can deduce the invasion risk of further spreading capability of a port, thus estimating the effect of *stepping-stone*. Taking both the incoming risk and outgoing risk into consideration, a biosecurity triggering method based on *s*-core rank is derived.

### Basis for our analysis

Our main idea is to provide biosecurity suggestion taking into consideration both the invaded risk of port and its ability of further spreading invaded species. For any port *j*, its invaded risk (i.e., *P*_*j*_(*Inv*)) is the accumulating invasion risks over all shipping routes passing through it [14], i.e.,

1 $$  P_{j}(\textit{Inv})=1-\Pi_{i}[1-P_{ij}(\textit{Inv})]  $$

where *P*_*ij*_(*Inv*) denotes the invasion risk from port *i* to *j*.

As we described in the introduction, a port’s ability of spreading invaded species should be analyzed from a global perspective. To that aim, we introduce a concept of the species invasion network (SIN). SIN can be depicted by a directed graph, namely *S*=(*V*,*E*,*W*), consisting of a set *V* of nodes (i.e., ports), a set *E* of edges (i.e., shipping routes) and the weight *w*_*ij*_∈*W* (*w*_*ij*_=*P*_*ij*_(*Inv*) of edge *e*_*ij*_∈*E*) denoting the invasion risk from port *i* to *j*.

According to the description above, both the invaded risk and SIN involve *P*_*ij*_(*Inv*)(*i*,*j*∈*V*). In this paper, we use the model proposed in [14] to calculate *P*_*ij*_(*Inv*)(*i*,*j*∈*V*). That is,

2 $$  \begin{aligned} P_{ij}(\textit{Inv})=1-\Pi_{e_{ij}}[1-P_{ij}(\textit{Alien})P_{e}(\textit{Intro})P_{ij}(\textit{Estab})] \end{aligned}  $$

In (2), *P*_*ij*_(*Alien*) is the probability that a native species in port *i* is non-native in port *j* [21, 22], which is inversely proportional to the shipping route distance between ports *i* and *j*; *P*_*e*_(*Intro*) denotes the survival probability of species entrained in ballast tanks and it increases with the total amount of ballast water; *P*_*ij*_(*Estab*) is the chance of species being able to live in the recipient port, which is affected by two main environmental factors: temperature and salinity. The detailed calculations of *P*_*ij*_(*Alien*), *P*_*e*_(*Intro*) and *P*_*ij*_(*Estab*) can be found in [14]. We omit them due to the limited length of paper.

**Fig. 2:**
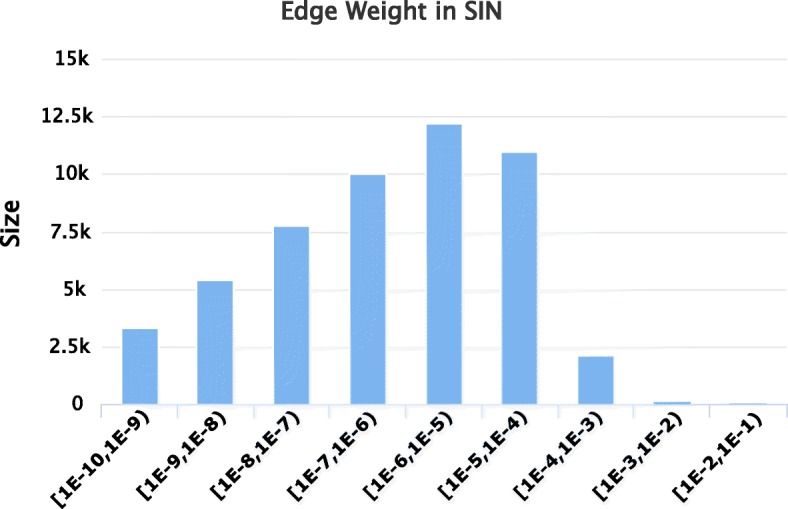
The constructed SIN

**Fig. 3:**
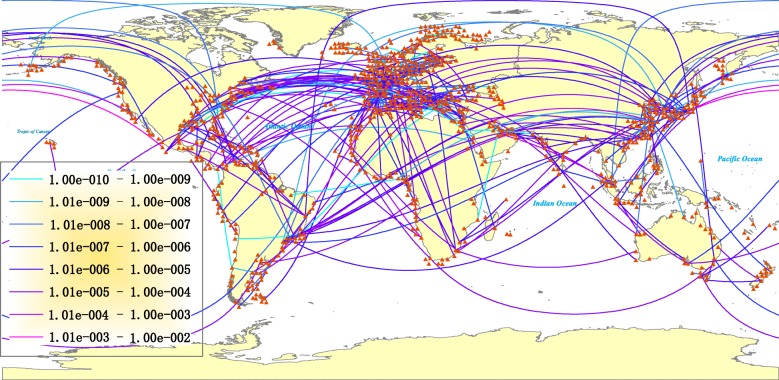
The distribution of edge weight in SIN

Taking advantage of the above models and the corresponding data, we can compute the invasion risk from one port to another and therefore obtain SIN. Figure 2 shows SIN computed according to our data. There are totally 34651918 weighted edges in the original SIN but only about 350 weighted edges are randomly selected to appear in Fig. 2. The distribution of edge weight in SIN is depicted in Fig. 3. Table 1 further lists the top 10 edges with the highest weights. As it can be seen in Table 1, the transportation between Singapore and Dubai, Seattle and Tokyo can incur more invasive risk. It is a remarkable fact that the bi-directed edges of Singapore-Dubai, Seattle-Tokyo and Klang-Dubai are listed in Table 1. The reasons for the result can be partly concluded as below: Singapore and Klang work as an important international maritime transport hub, serving the worldwide busiest trade routes; Dubai serves as a major transport hub for passengers and cargoes in the Middle East; the high weight between Seattle and Tokyo may refer to the strong economic connection between the United States and Japan.

**Table 1:** Top 10 edges with the highest weight in SIN

	Port of Departure	Port of Destination	Weights
1	Singapore	Dubai	0.013508770
2	Dubai	Singapore	0.010278908
3	Seattle	Tokyo	0.009086252
4	Tokyo	Seattle	0.008123248
5	Klang	Dubai	0.006145533
6	Bahrain	Klang	0.006009097
7	Le Havre	New York and New Jersey	0.004968214
8	Dubai	Klang	0.004948834
9	Tokyo	Manzanillo	0.004783012
10	Callao	Manzanillo	0.004436788

### *S*-core decomposition of SIN

The transmission power of a port stands for its potential to spread invaded species to others. Intuitively, the transmission power of each node is tightly related to the port’s topological property. To acquire the ports’ topological property, we use *s*-core decomposition to analyze the SIN. *S*-core decomposition, an extension of *k*-core decomposition [23], has shown its brilliant features in analyzing the structure of complex networks [19].

Through *k*-core decomposition, different subsets can be obtained, called *k*-cores. More specifically, let *d*_*i*_ be the out-degree of node *i* for an unweighted graph. The *k*-core of a graph consists of all nodes with degree *d*_*i*_>(*k*−1). Initially, 0-core consists of all nodes in the network. To obtain *k*-core, all nodes *i* with out-degree *d*_*i*_≤*k*−1 are iteratively removed from (*k*−1)-core. Thus, (*k*+1)-core is included in *k*-core. A *k*-shell is defined as a set of nodes in *k*-core that are not the members of (*k*+1)-core [19]. A node’s large degree and central position can be deduced by its large value of index *k*.

However, *k*-core decomposition is only suitable for graphs where the links are of uniform strength. To analyze SIN with heterogeneous edges, we employ *s*-core decomposition [19], which is a method extending *k*-core decomposition to weighted graphs. Firstly, we introduce *s*_*k*_-core decomposition to make the concept clearer. In *s*_*k*_-core decomposition, the weighted degree $d^{\prime }_{i}$ of a node *i* is defined as

3 $$  d'_{i}=[d_{i}^{\alpha}(\sum\limits_{j}^{d_{i}}w_{ij})^{\beta}]^{\frac{1}{\alpha+\beta}}  $$

where ${\sum \nolimits }_{j}^{d_{i}}w_{ij}$ is the sum over all its link weights and in our case, *w*_*ij*_=*P*_*ij*_(*Inv*); *α* and *β* are set to 1 according to [24]. The *s*_*k*_-core of a graph consists of all nodes with degree $d^{\prime }_{i}\geq s_{k}$. All *s*_*k*_-cores (*k*=0,1,2,…,*n*) are calculated by an iterative method. Initially, *s*_0_-core consists of all nodes in the network (*s*_0_= min*idi*′, *i*∈ all nodes). After iteratively remove all nodes *i* with weighted out-degree $d^{\prime }_{i}\leq s_{0}$, *s*_1_-core is obtained and then *s*_1_= min*idi*′, *i*∈*s*_1_-core. To extract *s*_*n*_-core, all nodes *i* with weighted out-degree $d^{\prime }_{i}\leq s_{n-1}$ are iteratively removed from *s*_*n*−1_-core and all nodes’ weighted degrees are recalculated for every removal. By this way, *s*_*n*_-core is obtained, where *s*_*n*_= min*idi*′, *i*∈*s*_*n*_-core. We reindex *s*_*k*_-core according to the rank of *s*_*k*_. Hence the decomposition of *s*_*k*_-core with a new index is just that of *s*-core. It is notable that the *s*_0_-core consists of all nodes and the *s*_*k*+1_-core is included in *s*_*k*_-core. A set of nodes in *s*_*k*_-core that are not the members of *s*_*k*+1_-core is called *s*-shell.

**Fig. 4:**
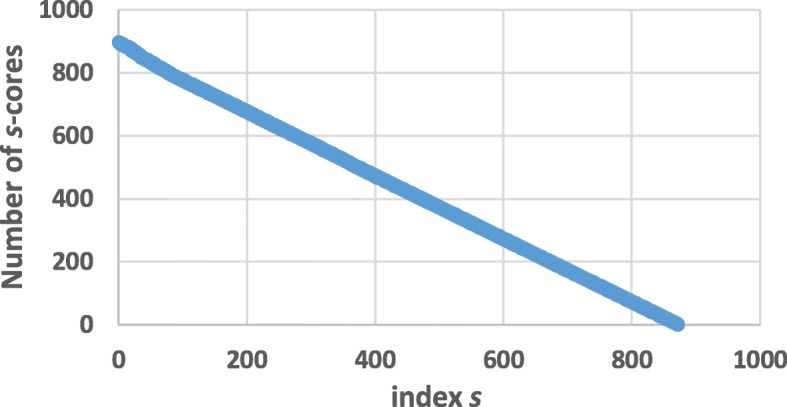
The *s*-cores in SIN

**Fig. 5:**
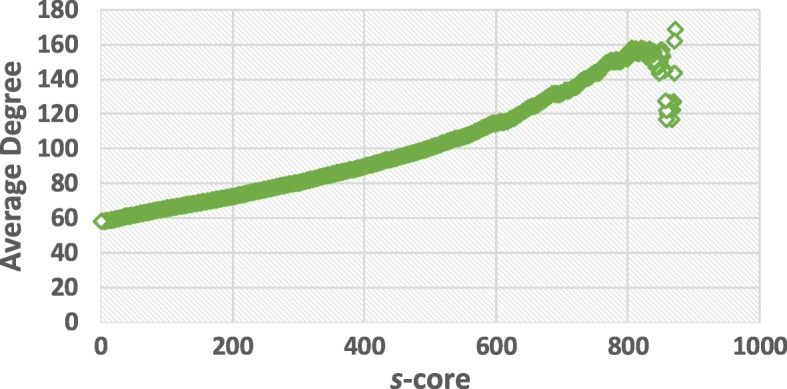
The average degree of different *s*-cores in SIN

**Fig. 6:**
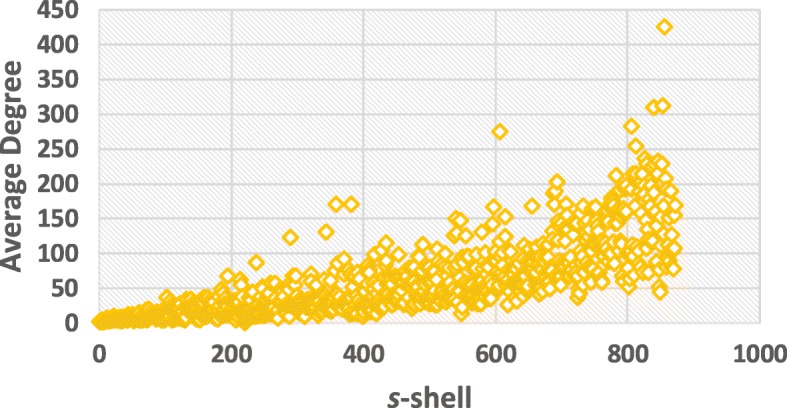
The average degree of different *s*-shells in SIN

**Fig. 7:**
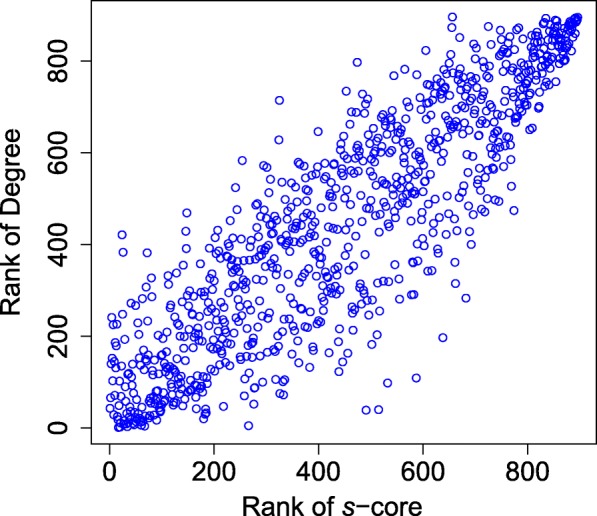
Correlation between the rank of *s*-core and that of the degree

According to the algorithm in [19], we can deduce *s*-shell of each node in SIN. Figure 4 indicates the number of *s*-cores in SIN with different *s*. Figures 5 and 6 illustrate the average degree of different *s*-cores and *s*-shells in SIN. Both figures show that the larger value of *s*, the higher average degree. Figure 7 further shows the correlation between the rank of *s*-core and that of the degree. The correlation analysis is executed through the Kendall rank correlation method [25], a statistic tool for estimating the similarity level between two ranks. Table 2 lists the top 10 ports ranked by their value of *s*-shell and Seattle, Tokyo and Callao are the top 3.

**Table 2:** Top 10 ports ranked by *s*-shell

Ranking	1	2	3	4	5
Port name	Seattle	Tokyo	Callao	Manzanillo	Incheon
*s*-shell	872	871	870	869	868
Ranking	6	7	8	9	10
Port name	Sydney	Kaohsiung	Brisbane	Perth	Qingdao
*s*-shell	867	866	865	864	863

## Results and discussion

Based on the bioinvasion risk of each port, biosecurity control and bioinvasion treatment can be triggered by our proposed biosecurity triggering method. The bioinvasion risk is evaluated by the invaded risk and invasion risk spreading capability of each port. The former is the incoming risk while the latter is the outgoing one. Therefore, we can trigger the corresponding bioinvasion control on a port j based on the following simple criterion:

4 $$  R(j)=\theta\widetilde{P}_{j}(\textit{Inv})+(1-\theta)\widetilde{s}(j) \geq T  $$

where *R*(*j*) is the bioinvasion risk of port *j*, and $\widetilde {P}_{j}(\textit {Inv})$ and $\widetilde {s}(j)$ are respectively the normalized *P*_*j*_(*Inv*) (the invaded risk of port *j* calculated using (1)) and the normalized *s*-shell value of that port; 0≤*θ*≤1 is the tradeoff weight. Smaller *θ* means more attention should be paid on the *stepping-stone* invasion and otherwise, the invaded risk should be obtained more concern; *T* is the given threshold helping to judge whether a bioinvasion treatment should be triggered. Larger *T* means the bioinvasion control starts up more hardly.

**Fig. 8:**
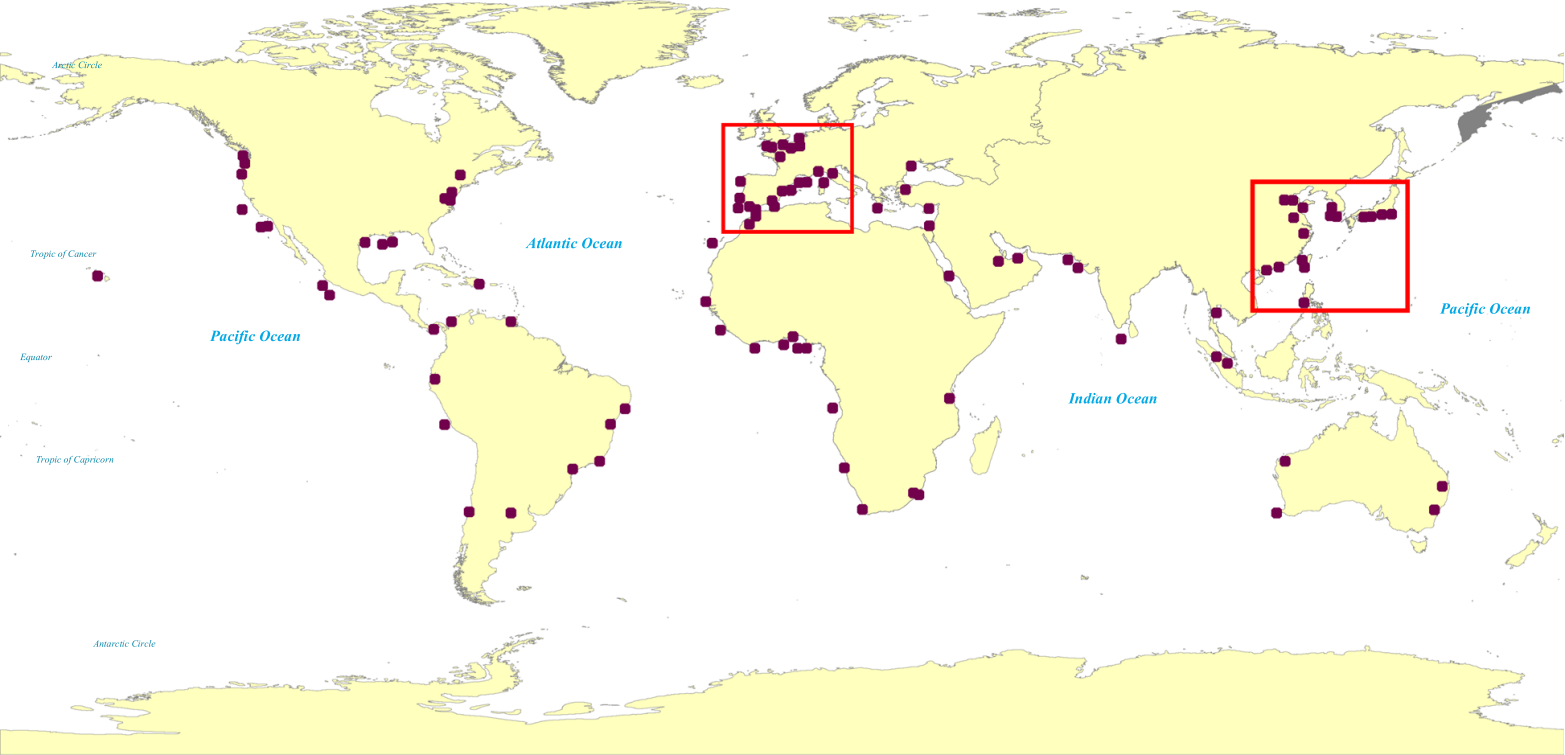
Top 100 ports with highest bioinvasion risk

Figure 8 shows the 100 ports whose values of $\theta \widetilde {P}_{j}(\textit {Inv})+(1-\theta)\widetilde {s}(j)$ are larger than others, where *θ*=0.5, meaning the incoming and outgoing risks are equally treated. From Fig. 8, compared to other regions, there are more bioinvasion risky ports concentrated in Western Europe (including the Western European margin and the Mediterranean) and the Asia-Pacific. These two regions are within the rectangles in Fig. 8. According to the statistical data [26], the number of recorded non-indigenous species has grown by 173% and 204% respectively in the Western European margin and the Mediterranean between 1970 and 2013. Furthermore, the Asia-Pacific has been identified as a source for many of non-indigenous species discovered elsewhere (especially the Asian clam, which is assumed perhaps the most invasive species worldwide) [27]. Hence, our analysis basically accords with the real-world marine bioinvasion status.

Table 3 further lists the top 10 ports ranked by their bioinvasion risk. The result shows Rotterdam is the most invasively risky port. Maybe just because of its high bioinvasion risk, a Ballast Detention Centre involving a transaction of some €100 million, was designed for Rotterdam harbor, which was the first custodial institution in the Netherlands to be contracted to a public-private partnership in Government spending on aquatic invasive species [28].

**Table 3:** Top 10 ports ranked by bioinvasion risk

Ranking	1	2	3	4	5
Port name	Rotterdam	Tokyo	Singapore	New York and New Jersey	Kaohsiung
Ranking	6	7	8	9	10
Port name	Dubai	Seattle	Manzanillo	Incheon	Colon

## Conclusions

To address the issue of aquatic bioinvasion, we propose a biosecurity triggering mechanism, where biosecurity controls should be triggered once the bioinvasion risk of a port is larger than a given threshold. The bioinvasion risk in our paper is measured according to both the invaded risk of a port and its ability of further spreading invaded species, which are calculated based on big data. We list 100 ports in the world that have the highest bioinvasion risk when the invaded risk and *stepping-stone* bioinvasion risk are equally treated. There are two bioinvasion risk intensive regions, namely the Western Europe (including the Western European margin and the Mediterranean) and the Asia-Pacific. According to the real-world data, the number of recorded non-indigenous species has grown rapidly in the Western European margin and the Mediterranean. Furthermore, the Asia-Pacific has been identified as a source for many of non-indigenous species discovered elsewhere (especially the Asian clam, which is assumed perhaps the most invasive species worldwide). Hence, our analysis basically accords with the real-world marine bioinvasion status. Topological importance (measured in light of betweenness and closeness) will be considered for designing a refined biosecurity triggering method in the future.

## Abbreviations

AIS: Automatic identification system
SFN: Species flow network
SIN: Species invasion network
